# Supplementary material for: The causal relationship between the human gut microbiota and pyogenic arthritis: a Mendelian randomization study
Source: Front Cell Infect Microbiol. 2024 Nov 26;14:1452480. doi: 10.3389/fcimb.2024.1452480 (PMC11629706; doi:10.3389/fcimb.2024.1452480)
Supplement: Supplementary file 1 [file DataSheet1.zip › mendelian test/ebi-a-GCST90027481.csv_leave_one_out.pdf]

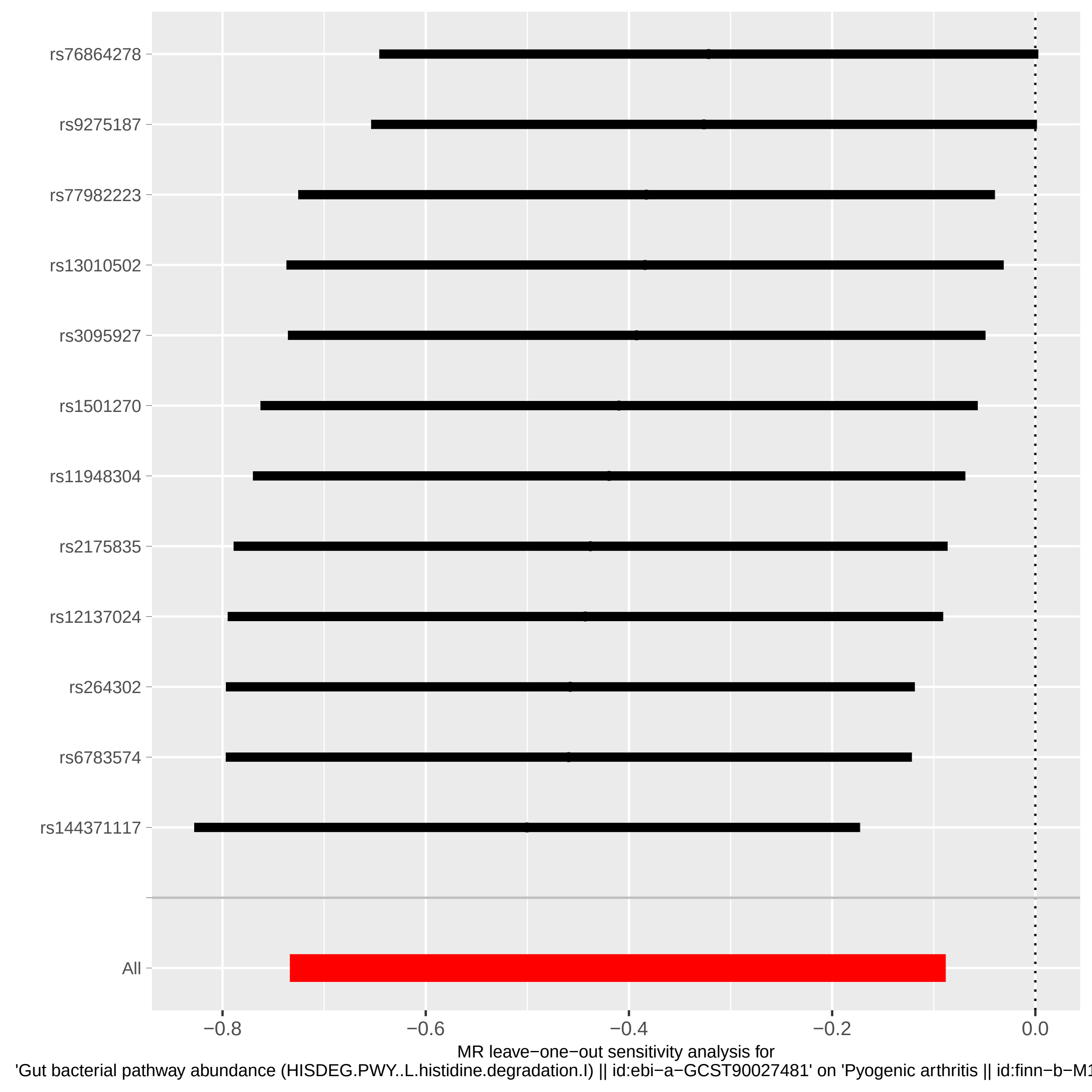
rs76864278
rs9275187
rs77982223
rs13010502
rs3095927
rs1501270
rs11948304
rs2175835
rs12137024
rs264302
rs6783574
rs144371117
All
−0.8
−0.6
−0.4
−0.2
0.0
MR leave−one−out sensitivity analysis for
'Gut bacterial pathway abundance (HISDEG.PWY..L.histidine.degradation.I) || id:ebi−a−GCST90027481' on 'Pyogenic arthritis || id:finn−b−M1
